# Genetic mechanisms in the repression of flowering by gibberellins in apple (*Malus* x *domestica* Borkh.)

**DOI:** 10.1186/s12864-019-6090-6

**Published:** 2019-10-16

**Authors:** Songwen Zhang, Christopher Gottschalk, Steve van Nocker

**Affiliations:** Department of Horticulture and Graduate Program in Plant Breeding, Genetics, and Biotechnology, Michigan State University, 390 Plant and Soil Science Building, 1066 Bogue St., East Lansing, MI 48824 USA

**Keywords:** Gibberellin (GA), Apple, Flowering, *GA2 OXIDASE* (*GA2ox*), *TERMINAL FLOWER 1 (TFL1)*, *FLOWERING LOCUS T* (*FT*)

## Abstract

**Background:**

Gibberellins (GAs) can have profound effects on growth and development in higher plants. In contrast to their flowering-promotive role in many well-studied plants, GAs can repress flowering in woody perennial plants such as apple (*Malus* x *domestica* Borkh.). Although this effect of GA on flowering is intriguing and has commercial importance, the genetic mechanisms linking GA perception with flowering have not been well described.

**Results:**

Application of a mixture of bioactive GAs repressed flower formation without significant effect on node number or shoot elongation. Using Illumina-based transcriptional sequence data and a newly available, high-quality apple genome sequence, we generated transcript models for genes expressed in the shoot apex, and estimated their transcriptional response to GA. GA treatment resulted in downregulation of a diversity of genes participating in GA biosynthesis, and strong upregulation of the GA catabolic *GA2 OXIDASE* genes, consistent with GA feedback and feedforward regulation, respectively. We also observed strong downregulation of numerous genes encoding potential GA transporters and receptors. Additional GA-responsive genes included potential components of cytokinin (CK), abscisic acid (ABA), brassinosteroid, and auxin signaling pathways. Finally, we observed rapid and strong upregulation of both of two copies of a gene previously observed to inhibit flowering in apple, *MdTFL1* (*TERMINAL FLOWER 1*).

**Conclusion:**

The rapid and robust upregulation of genes associated with GA catabolism in response to exogenous GA, combined with the decreased expression of GA biosynthetic genes, highlights GA feedforward and feedback regulation in the apple shoot apex. The finding that genes with potential roles in GA metabolism, transport and signaling are responsive to GA suggests GA homeostasis may be mediated at multiple levels in these tissues. The observation that *TFL1*-like genes are induced quickly in response to GA suggests they may be directly targeted by GA-responsive transcription factors, and offers a potential explanation for the flowering-inhibitory effects of GA in apple. These results provide a context for investigating factors that may transduce the GA signal in apple, and contribute to a preliminary genetic framework for the repression of flowering by GAs in a woody perennial plant.

## Background

Gibberellins (GAs) are a class of phytohormones that have profound effects on many aspects of plant growth and development. GAs are required for such diverse functions as mobilization of starch reserves in barley aleurone cells, seed germination in response to stratification or light signals, and promotion of hypocotyl and stem elongation in darkness or shade [1]. GAs also have a well-documented role to promote flowering in long-day rosette plants such as the research reference *Arabidopsis thaliana* (Arabidopsis) [1]. At the cellular level, GAs have been best characterized as promoting cell elongation, but whether this mechanism drives all of its organismal functions remains unclear [2].

GAs comprise a wide variety of diterpenoid compounds sharing a 19- or 20-carbon tetracyclic structure. To date, 136 molecularly distinct GAs have been identified, designated as GA_1_ - GA_136_. However, only GA_1_, GA_3_, GA_4_ and GA_7_ are known to have biological activity in higher plants [3]. The initial GA in the biosynthetic pathway, GA_12_, is synthesized from GGPP (trans-geranylgeranyl diphosphate) in the chloroplast, via a series of enzymes including CPS (*ent*-copalyl diphosphate synthase), KS (*ent*-kaurene synthase), KO (*ent*-kaurene oxidase), and KAO (*ent*-kaurenoic acid oxidase). GA20ox (GA20-oxidase) is responsible for the removal of C20 (carbon 20) to form C19-GAs (GA_9_ and GA_20_), and further oxidation by GA3ox (GA3-oxidase) results in the final formation of the bioactive GAs [3]. The GA2ox (GA2 oxidase) group of enzymes catalyze the oxidation of both bioactive (except GA_3_) and inactive GAs, and thus represent an important mechanism for catabolism of GAs and the resetting of GA signaling [4]. Additional mechanisms for inactivation of bioactive GAs include oxidation by the EUI/ELA class of cytochrome p450 monooxygenases, which target non-13-hydroxylated GAs such as GA_4_ [5–7].

GAs can be transported across the plasma membrane by several members of the Nitrate transport 1/Peptide transporter family (NPF), which also participate in transporting a wide range of other substrates [8]. At least NPF2.10, NPF3.1 and NPF4.1 have been shown to transport bioactive GAs (GA_1_, GA_3_, and GA_4_) [9–12]. In Arabidopsis, NPF3.1 can also transport biologically inactive GAs (GA_9_, GA_12_, and GA_20_) and the GA catabolite, GA_8_ [12].

At the molecular level, the GA signaling pathway comprises three elements: (1) GID1 (GIBBERELLIN-INSENSITIVE DWARF 1) proteins, which act as GA receptors, (2) a class of transcriptional effector proteins designated DELLA, and (3) a set of transcription factors that initiate changes in downstream GA response-associated genes [13]. In all contexts studied so far, binding of GA to GID1-type receptors promotes interaction between GID1 and a DELLA protein, which then triggers targeting of the DELLA protein by the SCF^GID2/SLY1^ complex [13, 14]. SCF^GID2/SLY1^ is an E3 ligase that directs ubiquitination of the targeted DELLA proteins, hence initiating their degradation by the 26S proteasome [14]. DELLA proteins can influence transcription by directly interfering with the function of transcription factors, or by activating transcription directly [15].

Plants have several mechanisms to maintain GA at a certain level (GA homeostasis). In plant tissues, GA homeostasis can be maintained through both repression of GA biosynthesis and promotion of GA catabolism. Repression of biosynthesis (feedback regulation) is known to be mediated by decreased expression of the GA biosynthetic genes, *GA20OX* and *GA3OX*, whereas promotion of catabolism (feedforward regulation) results from increased expression of the GA catabolic genes, *GA2OX* [16–19]. GA homeostasis has also been shown to be achieved via feedforward regulation of DELLA genes, and feedback regulation of receptor and transporter genes [12, 20, 21].

In Arabidopsis, GAs promote floral induction by upregulating the floral meristem identity genes *SOC1* (*SUPPRESSOR OF OVEREXPRESSION OF CO 1*) and *LEAFY* (*LFY*) [22–24]. The regulation of *LFY* is at least in part dependent on binding of the GA-MYB transcription factor, AtMYB33, to a regulatory element within the *LFY* promoter [25]. GA can also promote floral induction by regulating *SPL* (*SQUAMOSA PROMOTER BINDING–LIKE*) genes, which encode transcription factors that positively regulate the expression of *SOC1* and *LFY* [26]. In addition, GA promotes the expression of the *FT* (*FLOWERING LOCUS T*) gene in Arabidopsis leaves [27]. *FT* encodes the protein that is believed to be the florigen and transported from leaf to shoot apical meristem [28, 29]. Ultimately, increased expression of *SPL*, *SOC1*, *LFY* and *FT* promote expression of *APETALA1* (*AP1*) and other genes that direct flower formation [30].

The promotion of flowering by GAs in Arabidopsis is in contrast to the generally repressive effects of GAs on flowering in some woody perennial plants including apple [31, 32], peach [33], citrus [34], and grapevine [35]. Domesticated apple trees have a biennial (two-year) flowering cycle, such that flowers are initiated during the growing season in the first year and complete development and bloom in the growing season of the second year. Application of either GA_3_ or a mixture of GA_4_ and GA_7_ (GA_4 + 7_) to whole trees during the period of floral induction in the first year can reduce bloom the second year [32, 36–40]. When carried out in commercial operations, the reduction in fruit load enhances fruit size and quality [41–43].

Although the repressive effect of GAs on flowering in apple has great commercial importance, there is little understanding of the fundamental mechanisms. This lack of knowledge has limited the development of more efficient methods to control flowering in apple, and the breeding of new cultivars with more desirable flowering traits [44–46]. It has been hypothesized that the effect of GAs in apple may be mediated through inhibiting activity of cytokinins (CK), which have been shown to promote floral initiation [47, 48], but the molecular relationship between GA and CK in apple has not been well studied. Underlying this deficit in knowledge is the lack of fundamental information about GA-responsive gene expression in apple and other woody perennial plants. Ultimately, the repression of flowering by GA is expected to be driven by changes in the expression of those apple genes that have key roles in flowering. Haberman et al. [49] found that GA applied to apple early in the growing season (~ 30 DAFB) led to increased expression of the *MdTFL1–2* gene, one of two *TFL1* homologs in apple, late in the season when flowers would be forming. Previous studies found that apple *TFL1* gene(s) repressed flowering when expressed ectopically in Arabidopsis [50], while antisense expression of these genes in apple led to abbreviation of the juvenile phase and early flowering [51]. However, it was unclear whether the increased expression of *MdTFL1–2* late in the season observed by Haberman et al. [49] reflected a direct action of GA on the *MdTFL1–2* gene, or rather if *MdTFL1–*2 expression was an indirect result of floral repression. This is conceivable, because in Arabidopsis, *TFL1* is negatively regulated by *LFY* and *AP1* [52].

In this study, we documented the transcriptional network influenced by exogenous GA in the apple shoot apex under conditions that repress floral induction, as a first step to understand the flowering-repressive role of GA in woody perennial plants.

## Results

### Repression of flower formation by GA

**Fig. 1:**
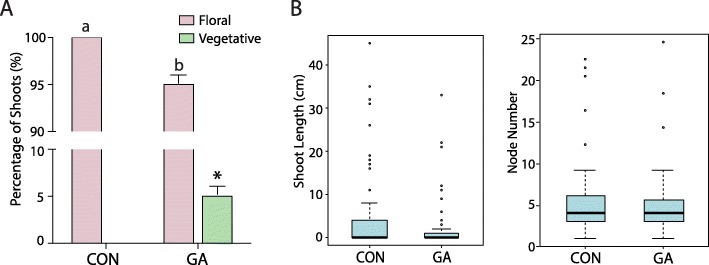
GA_4 + 7_ treatment led to significant repression of flowering without altering shoot architecture. **a** Percentage of floral and vegetative shoots initiating from spurs on control (CON) and GA-treated trees. Shoots were evaluated during early spring the year following the experiment. Letters and the asterisk above the bars indicate statistically significant differences between GA-treated trees and control (analysis of variance, *p* < 0.05). **b** Length and node number of bourse shoots initiated from spurs on control and GA-treated trees. Shoots were evaluated at the end of the growing season. Boxes represent the median, 25th and 75th percentile values; error bars show outlier boundaries (inter-quartile range); bubbles above represent outliers

Like many commercial apple cultivars, ‘Gala’ produces flowers in an inflorescence that forms terminally on short shoots called spurs. Growth in the subsequent season originates from a dominant apical bud subtending the inflorescence. These new shoots, called bourse shoots, typically produce four to six leaves in a rosette and terminate with inflorescence primordia, reiterating the previous season’s structure. Alternatively, bourse shoots may remain vegetative, and occasionally these elongate to form lateral branches. To demonstrate the influence of GA on flowering and generate material for molecular analyses, we applied a commercial formulation of a GA_4_/GA_7_ mixture as a foliar spray to ‘Fulford Gala’ trees, approximately 35 days after full bloom (DAFB). This treatment was timed to coincide with early floral induction, based on published data for this cultivar [53, 54] and our preliminary observations (not shown). All flowers were removed from these trees just before bloom, to avoid potential influences of bioactive GAs generated by developing fruit [55]. To minimize unanticipated effects on growth and development, we used a relatively low (200 ppm) concentration of GAs. Consistent with previous observations on the effect of GAs in apple, this treatment led to subtle, but significant, repression of flowering (Fig. 1a), without significantly altering shoot architecture, as evaluated by quantifying shoot length and node number (Fig. 1b).

### Gene expression within the bourse shoot apex

To assess the effect of exogenous GAs on the transcriptional landscape within the bourse shoot apex in the context of flowering, we carried out sequencing-based transcriptional profiling (RNA-seq) of the bourse shoot apex, which contains the apical meristem, subtending leaf primordia, and underlying tissues, including pith and vasculature. Collections were made at 1, 2, 5, and 15 days after GA treatment (DAT). RNAs were subjected to sequencing using the Illumina HiSeq platform and 101-base, paired-end reads. The resulting ~ 379 million read pairs (Additional file 1: Table S1) were aligned to a recently released, *Malus* x *domestica* genome sequence (GDDH13) [56] and assembled into transcript models (Additional file 2).

The exhaustive depth of our transcriptional data and recent availability of a high-quality apple genome sequence provided the opportunity to document genes expressed in the apple bourse shoot apex with high resolution and accuracy. When analyzed collectively, this data supported the expression of 84,732 distinct transcripts (Additional file 3) from 40,487 loci (Additional file 4). About 81% of these loci (32,794) had been previously annotated as protein-coding or noncoding genes in the apple reference genome [56] (Additional file 4). Among the remaining 7693 loci, 6952 would generate transcripts showing no overlap with exons of annotated reference genes (Additional file 5: Table S2). As anticipated, most of these showed strong homology to genes that had previously been identified from apple, but that were absent from the reference genome annotation (Additional file 5: Table S3). Transcripts from 970 additional loci had not previously been identified in apple, although they showed significant homology with sequences that had been described previously in other plant species (Additional file 5: Table S3). A further 1291 loci lacked significant homology to any sequence in current public databases, including the draft *Malus* x *domestica* genome sequence (Additional file 5: Table S4). Compared with the apex transcriptome as a whole, these novel unknown loci were weakly expressed (median FPKM < 1), and generated relatively short transcripts (median length 382 bp) (Additional file 5: Table S4). Because these transcripts were modeled on sequence reads that were aligned with the reference genome, they likely represent authentic apple loci, rather than contamination of the sequence dataset by exogenous biota. The reference genome contained 16,694 loci that were not supported by our data from the apple bourse shoot apex (Additional file 5: Table S2), consistent with the broad mRNA sequence representation used in annotation of the GDDH13 reference genome [56], and the highly restricted tissue source used in this study. A revised feature coordinate map of the GDDH13 reference genome including both the previously and newly annotated loci is presented as Additional file 6.

### Transcriptional response of GA signaling components

**Fig. 2:**
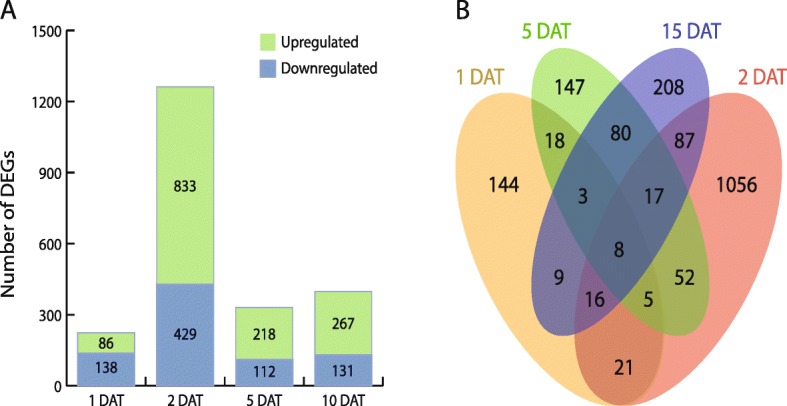
Visualization of numbers of genes expressed differentially in response to GA. **a** Number of significantly upregulated and downregulated genes at each time point (q < 0.05). **b** Venn diagram showing the number of differentially expressed genes at one or multiple time points

By comparing sequence representation for transcript models in shoot apices from GA-treated vs. control trees, we identified genes and transcripts whose abundance was influenced by GAs. We found 1476 genes that showed a significant transcriptional response to GA treatment at one or more of the four time points (Fig. 2 and Additional file 7: Table S5). Analysis of this subset of gene for potential ontology revealed overrepresentation for several bioprocess terms, including two related to GA: ‘gibberellin biosynthetic process’ and ‘response to gibberellin’. An independent analysis of potential molecular interactions (KEGG pathway) revealed overrepresentation for two pathway terms associated with GA metabolism and signaling: ‘diterpenoid biosynthesis’ and ‘plant hormone signal transduction’ (Additional file 8: Tables S6 and S7). These findings are consistent with the expected disruption of GA signaling in response to exogenous bioactive GA.

**Fig. 3:**
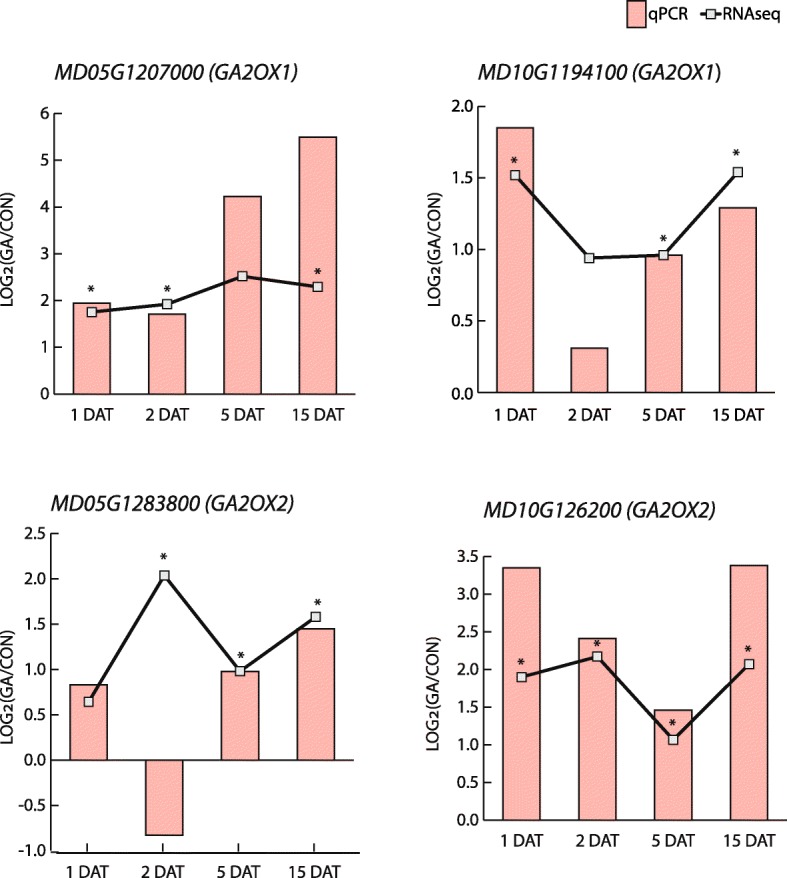
Four *GA2OX* genes were expressed to significantly higher levels in GA-treated shoot apices. Lines and significance (asterisk *, *p* < 0.01) were based on RNA-seq data, while bars were based on RT-PCR data. Log_2_(GA/CON) denotes Log_2_(Fold Change)

**Fig. 4:**
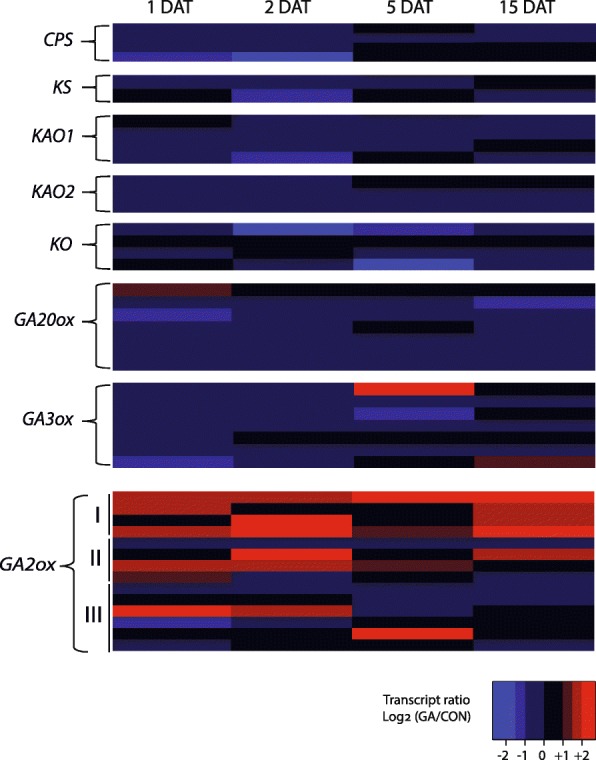
Expression of GA biosynthetic genes, and GA catabolic genes *GA2OX* in response to GA. Biosynthetic genes include *CPS*, *KS*, *KAO*, *KO*, *GA20OX*, and *GA3OX*. Color key on the bottom right indicates high positive (red) and negative (blue) fold difference in expression between GA-treated and non-treated plants. For GA2OX genes, members of Classes I, II, and III are indicated

Of the apple GA-regulated genes that could be classified by GO (Gene Ontology) bioprocess and KEGG pathway enrichment analyses, 29 were included in at least one of these GA-associated groups (**not shown**). These genes collectively represented the breadth of known genes associated with GA metabolism and signaling. Two genes closely related to *KS1* and *GA20OX*, and thus potentially participating in biosynthesis, were significantly downregulated at 2 DAT and 15 DAT, respectively (Table 1). In contrast, four distinct *GA2OX* homologs showed increased expression at least at three of the time points (Table 1 and Fig. 3). This opposing influence of GA on *GA20OX* and *GA2OX* gene expression in apple is consistent with that previously observed by Haberman et al. [49], and is suggestive of GA feedback and feedforward regulation. To document this in more detail, we identified all potential GA biosynthetic (*KAO*, *KO*, *CPS*, *GA20OX*, *GA3OX*) and catabolic (*GA2OX*) genes in our dataset, based on sequence homology with functionally characterized genes from Arabidopsis (Additional file 9: Table S8), and estimated their expression at all four time points following GA application, relative to non-treated samples (Fig. 4). This showed that GA_4 + 7_ generally repressed the expression of GA biosynthetic genes. In contrast, GA_4 + 7_ generally promoted expression of an assortment of *GA2OX* genes. This was most apparent for those *GA2OX* genes representing Class I and Class II enzymes, which target C19-GAs and thus are capable of inactivating bioactive GAs (Fig. 4). Inspection of the GA-responsive genes that were not annotated with GA-related ontology terms identified a homolog of *EUI/ELA1*, which encodes a GA_4_ catabolic enzyme and represents another GA deactivation pathway [5–7]. This *EUI*/*ELA1*-like gene was moderately upregulated at 15 DAT (Additional file 7: Table S5).

**Table 1:** GA-associated differentially expressed genes

Terms	Gene ID	Arabidopsis Homolog ID	Gene	Log2(Fold Change)^a^			
				1 DAT	2 DAT	5 DAT	15 DAT
Gibberellin biosynthetic process							
	MD10G1215700, MD10G1215900, MD10G1216200	AT1G79460.1	GA2, KS, ATKS1		−1.13		
	MD00G1003600	AT5G51810.1	GA20OX2, AT2353				−1.12
	MD05G1283800	AT1G30040.1	ATGA2OX2, GA2OX2		2.04	0.98	1.58
	MD10G1262000	AT1G30040.1	ATGA2OX2, GA2OX2	1.93	2.09	1.15	2.11
	MD10G1194100	AT1G78440.1	ATGA2OX1, GA2OX1	1.56	0.92	0.81	1.54
	MD05G1207000	AT1G78440.1	ATGA2OX1, GA2OX1		1.99	2.1	2.48
	MD14G1234300	AT1G02400.1	ATGA2OX4, ATGA2OX6, DTA1				−2.86
Response to gibberellin							
	MD15G1180500	AT1G14920.1	GAI, RGA2		1.09		
	MD03G1273300	AT3G63010.1	ATGID1B, GID1B	−1.45	−3.62		−1.41
	MD11G1296000	AT3G63010.1	ATGID1B, GID1B	−1.02	−2.22		−0.84
	MD04G1212400	AT5G27320.1	ATGID1C, GID1C		−1.23		−0.79
	MD17G1260700	AT3G03450.1	RGL2		0.88		
Plant hormone signal transduction							
	MD08G1091700	AT4G24210.1	SLY1		−1.74		−1.51
	MD15G1075800	AT4G24210.1	SLY1		−1.2		−0.89

^a^log_2_(Fold Change) is the log_2_ value of expression level in GA-treated buds versus that in control. Only significantly different values are listed in table

**Fig. 5:**
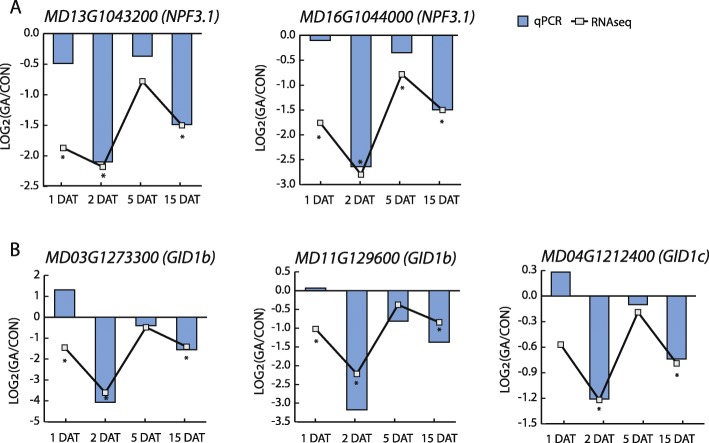
Downregulation of GA transporter- and receptor-like genes. **a** GA transporter-like genes, *NPF3.1*; **b** GA receptor like genes, *GID1*. Lines and significance (asterisk *, *p* < 0.01) were based on RNA-seq data, while bars were based on RT-PCR data. Log_2_(GA/CON) denotes Log_2_(Fold Change)

In addition, GA strongly downregulated the expression of 2 GA transporter-like genes, related to NPF3.1 (NITRATE TRANSPORTER AND PEPTIDE TRANSPORTER FAMILY 3.1) (Fig. 5a) and 3 GA receptor-like genes, related to GID1 (Fig. 5b). Interestingly, all five of these genes showed a similar temporal response to GA: little or no response at 1 DAT, strongest downregulation at 2 DAT, subtle downregulation at 5 DAT, and moderate downregulation at 15 DAT. This finding suggests a common but complex mechanism for regulation of these genes. GA also downregulated two genes homologous to Arabidopsis *SLY1* (or rice *GID2*), whose product is a component of the E3 ligase that targets DELLA proteins for degradation (Table 1). In contrast, two genes encoding potential DELLA proteins, *GAI* (*GIBBERELLIC ACID INSENSITIVE*) and *RGL2* (*REPRESSOR OF GA-LIKE 2*), were upregulated by GA at 2 DAT (Table 1). In summary, the expression of genes participating not only in GA metabolism, but also various signaling steps, were responsive to GA.

In addition to the GA pathway, exogenous GA also altered expression of genes with potential functions in other hormonal signal transduction pathways (Additional file 8: Table S7; Additional file 10: Table S9), including the CK, auxin, abscisic acid (ABA) and brassinosteroid pathways. Specifically, GA persistently downregulated the expression of two genes encoding homologs of AHP1, a histidine phosphotransfer protein connecting CK receptors with CK-mediated transcription (Additional file 10: Table S9). Consistent with its antagonism with ABA signaling in various developmental events, GA repressed the expression of potential key components in ABA signaling, which includes its receptors, as well as positive and negative signal mediators (Additional file 10: Table S9). GA was also associated with diminished expression of three genes with potential roles in brassinosteroid biosynthesis (Additional file 10: Table S9). In contrast to its repressive role in regulation of CK-, ABA- and brassinosteroid-related genes, GA widely upregulated the expression of potential components of auxin signaling, including an auxin receptor and negative and positive regulators (Additional file 10: Table S9). Taken together, these results suggest that, in the shoot apex at this developmental stage, GA signaling interacts extensively with other hormonal signaling pathways.

#### Transcriptional response of potential flowering genes

An unresolved question is the identity of flowering genes that may mediate the repressive effect of GA on flowering in apple. The timing of our analysis - between 35 and 50 DAFB, precedes the seasonal time at which floral differentiation is typically observed in this cultivar under these conditions (not shown). Consistent with this, GO terms related to flower formation were not enriched within the set of GA-responsive genes. An inspection of the set of GA-responsive genes identified four with annotations related to floral initiation. Two of these were closely related to *AP1*, and based on nucleotide sequence may represent the counterpart(s) of *MdMADS2* and/or *MdMADS5*, which had previously been shown to promote flowering when expressed heterologously in transgenic Arabidopsis or tobacco [57, 58]. Consistent with a presumed role promoting flowering, both of these *AP1*-like genes were expressed to significantly lower levels in GA-treated trees relative to controls, but this difference was seen only at a single time point (2 DAT) (Additional file 7: Table S5).

**Fig. 6:**
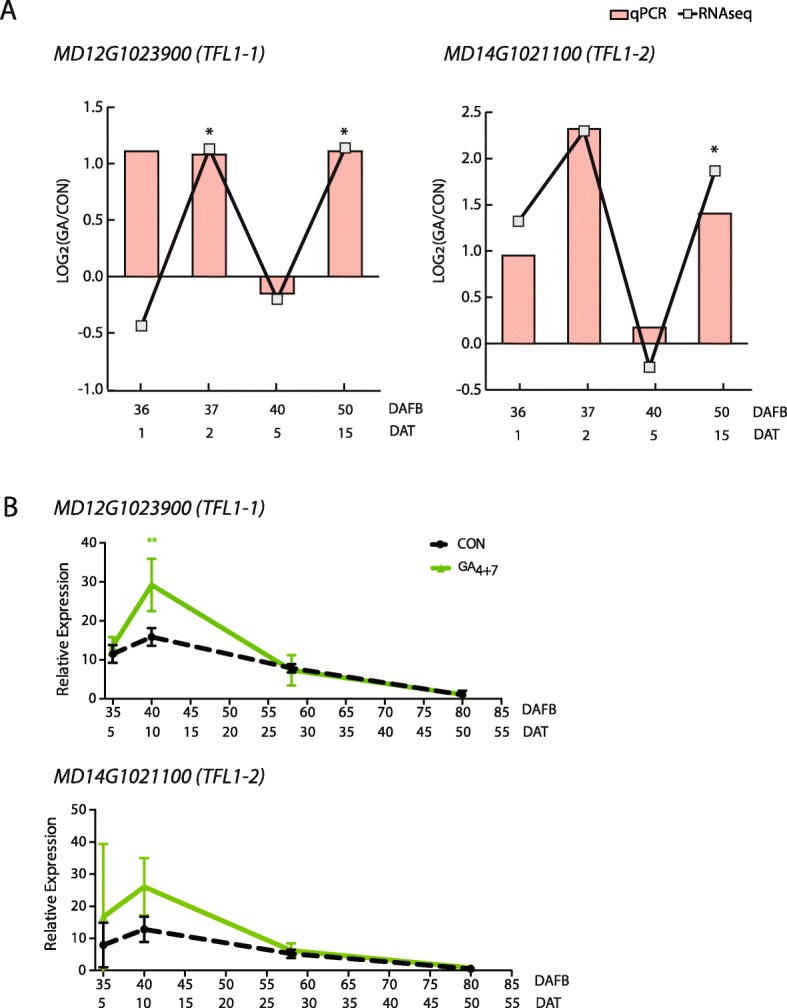
Upregulation of two apple *TFL1*-like genes in response to GA. **a** RNA-seq (line) and qPCR (column) measurement for ‘Fulford Gala’. Asterisk (*) denotes the difference between the expression of the gene in GA-treated sample and that in control was significant (p < 0.01). Log_2_(GA/CON) denotes Log_2_(Fold Change). **b** QPCR measurement for ‘Brookfield Gala’. Green line represents relative expression in GA_4 + 7_-treated bourse shoot apex, and black dashed line represents CON. Double asterisks (**) denote significant difference between CON and GA (p < 0.01). Error bars represent standard deviations. Double time scales are given on Y axis: DAFB, days after full bloom; DAT, days after treatment

The remaining GA-responsive, annotated flowering genes were *MdTFL1–1* and *MdTFL1–2*. Both genes were expressed to substantially higher levels in GA-treated trees compared with non-treated trees at 1, 2 and 15 DAT (Fig. 6a). *MdTFL1–2* was previously reported to be expressed to higher levels in the shoot apex of GA-treated vs. non-treated trees, but only very late in the growing season when flowers would be forming [49]. To further investigate a potential rapid influence of GA_4 + 7_ on *MdTFL1–1/MdTFL1–2* gene expression, we carried out a supplementary and independent transcriptional analysis. Five-year-old ‘Brookfield Gala’ trees, a clone of ‘Gala’ characterized by strongly striped fruit, were treated with a single application of 200 ppm GA_4 + 7_ at 30 DAFB, and gene expression was evaluated in the shoot apices of treated and non-treated control trees at 5, 10, 28 and 50 days following treatment, corresponding to 35 to 80 DAFB. In non-treated trees, both *MdTFL1–1* and *MdTFL1–2* showed a slight increase in expression between 35 and 40 DAFB, and steadily decreasing expression thereafter (Fig. 6b). Neither *MdTFL1–1* nor *MdTFL1–2* appeared to be expressed strongly at 80 DAFB. However, both *MdTFL1–1* and *MdTFL1–2* were expressed to significantly higher levels in GA-treated trees at 10 DAT (Fig. 6b), consistent with the immediate, GA-associated promotion of expression seen in the initial experiment.

We analyzed our datasets for expression of apple counterparts of four components linking GA with flowering in Arabidopsis - *GAMYB33*, *SOC1*, *FT,* and *LFY*. Although the GDDH13 apple reference genome includes clear homologs of these genes, and corresponding transcriptional models suggest that several were expressed in the apex, our data did not support their regulation by GA within the 15-d time frame at this developmental stage (Additional file 9: Table S8 and not shown). We examined *FT* more closely, because both of the two copies of *FT* in apple (*MdFT1*, *MdFT2*) have been shown previously to promote flowering when expressed ectopically in transgenic apple [59]. Consistent with previous observations [59], our analysis of RNA-seq read counts (Additional file 11: Figure S1a), and qPCR analysis (Additional file 11: Figure S1b), suggested that, in the shoot apex of non-treated trees, *MdFT1* was expressed moderately, whereas *MdFT2* was expressed at only very low levels. Similar to the results of the initial transcriptional profiling analysis, expression of *MdFT1* was unaffected by the GA treatment in the apex at any time point evaluated (Additional file 12: Figure S2).

#### Identification of transcription factors and *cis*-elements potentially participating in GA-responsive gene expression

We anticipate that specific, yet-unidentified transcription factors participate in promoting or repressing gene expression in response to GA, and that one or more of these may directly regulate flowering genes. We identified a total of 136 transcription factor-like genes as GA-responsive at at least one time point. These genes represented 32 diverse and well-studied transcriptional regulator classes, including AP2, bHLH, bZIP, GRAS, HD-ZIP, MADS, MYB, WRKY, and YABBY (Additional file 13: Table S10). Using a GO bioprocess enrichment analysis for GA-responsive genes in Arabidopsis that encode transcription factors, we identified 16 GO categories showing significant enrichment in our data set. Among these, there were four categories that showed exceptional enrichment (≥ 5-fold enrichment; *p* value <1E-04): ‘response to gibberellin’, ‘cell differentiation’, ‘ethylene-activated signaling pathway’, and ‘response to abscisic acid’ (Additional file 14: Figure S3).

There were nine transcription factor-encoding genes in the category ‘response to gibberellin’, including the two *GAI*/*RGL2*-like genes discussed above. Two *TCP14* (*TEOSINTE BRANCHED1/ CYCLOIDEA/PROLIFERATING CELL FACTOR 14*)-like genes were significantly upregulated by GA at 2 DAT (Additional file 7: Table S5). TCP14 is a direct target of DELLA proteins, and participates in GA-mediated seed germination [60]. Two homologs of Arabidopsis *KNAT1* (*KNOTTED-LIKE FROM Arabidopsis thaliana*) were also significantly upregulated by GA (Additional file 7: Table S5). *KNAT1* encodes a class I KNOX transcription factor, which regulates GA metabolic genes in the shoot apical meristem and promotes meristem function [61].

GA-responsive gene expression is expected to be mediated at least partly through the binding of transcription factors to gene regulatory *cis*-elements. We analyzed the promoter regions of the full set of GA-responsive genes for oligonucleotide motifs that were significantly enriched relative to the corresponding promoter regions of all expressed genes. We used a complementary approach to search for ungapped motifs of significantly overrepresented sequence patterns (see *Methods*). Motifs identified through these methods were compared with known *cis*-elements identified experimentally in previous studies by deletion analysis or by direct protein-nucleic acid binding [62, 63]. This approach resulted in the identification of potential binding sites for MYB1 (MYELOBLASTOSIS 1), WRKY14 (WRKY TRANSCRIPTION FACTOR 14), CDF5/CDF3 (CYCLING DOF FACTOR 5 or 3), bZIP48/bZIP44 (BASIC LEUCINE ZIPPER 48 or 44), and REM1 (REPRODUCTIVE MERISTEM 1) at 2 DAT (Additional file 15: Figure S4). One motif enriched at 5 DAT is the potential binding site for the homeobox transcription factor LMI1 (LATE MERISTEM IDENTITY 1) (Additional file 15: Figure S4), whose Arabidopsis homolog is characterized as a floral meristem identity gene and can interact with LFY. One enriched at 15 DAT is the potential binding site for the MYB-like transcription factor RVE (REVEILLE 1) (Additional file 15: Figure S4), whose Arabidopsis homolog is involved in hypocotyl growth by promoting auxin production [64].

To gain further insight into transcriptional networks mediating GA repression of flowering, we compared the set of GA-responsive transcription factors with the list of potential transcriptional cis-elements identified as enriched within all GA-responsive genes. We identified a single overlap: a *bZIP44* homolog was identified both as a GA-responsive gene, and as a potential cognate factor for a *cis*-element found within GA responsive genes. In Arabidopsis, bZIP44 has been studied as a transcriptional activator of a GA-responsive mannanase gene during seed germination [65]. Both the GA-associated difference in expression and the cis-element enrichment were significant at the same time point, 2DAT, suggesting that the association is functionally relevant.

## Discussion

The molecular mechanisms associated with the repression of flowering in apple and other woody perennial plants by bioactive GAs have not been extensively studied. Within the past decade, the availability of genomic data for apple has allowed for a molecular approach to studying developmental processes in this important horticultural crop, and several previous efforts have examined the involvement of a limited set of well-known candidate genes in flowering. Here, we carried out an unbiased, high-resolution transcriptional profiling of apple shoot apices subject to the flowering-repressive effects of GA, leveraging a reproducible field model and recently released, high-quality genome sequence.

Unlike its seemingly polar effects on floral induction across plant species, GA is well known to universally promote shoot elongation. A trivial explanation for its repressive effects on flowering in apple is that, by promoting elongation of the bourse shoot, GA decreases the numbers of condensed spur structures, on which flowers are initiated [31, 40]. In this study, however, we found no significant effect of GA on shoot architecture. This strongly argues that the effect is more direct.

At the molecular level, GA impacts gene expression within two very different arenas. Within the first, the expression of genes involved in the biosynthesis and catabolism of GAs are quickly adjusted to moderate GA concentration. These so-called feedback and feedforward mechanisms of regulation are important to maintain responsiveness to the hormone, thereby allowing the plant to use the GA signaling pathway to continuously adjust its growth and development in response to endogenous and environmental cues. Within the second, GA influences the expression of genes that direct growth and developmental processes. This study provides insight into both arenas of action within the apple shoot apex. The rapid and robust upregulation of four *GA2OX* genes in the apple shoot apex is consistent with findings of Fan et al. [31] in a different apple cultivar and implies participation of these genes in feedforward regulation. In rice and Arabidopsis, bioactive GA can also be catabolized through a pathway involving the EUI/ELA class of cytochrome P450 monooxygenase [5–7], and our observation that an apple *ELA1*-like gene is also GA responsive suggests that this protein may participate in feedforward regulation as well.

GA repressed expression of various GA signaling components, including GA transporters, receptors and DELLA repressors. NPF3.1 was identified as a GA importer in Arabidopsis roots [12], and appears to be well conserved among various plant lineages [66]. NPF3.1 shows a high affinity to GA_4_ compared to GA_3_ and GA_1_, and its expression is under the feedback regulation of GA such that GA application represses the expression of *NPF3.1*, and thus inhibits GA uptake [12], which is consistent with our finding in this study. The finding that GA application was also associated with decreased expression of three *GID1-*like receptor genes is in agreement with a previous study that concluded that GA homeostasis may be achieved not only via the regulation of GA metabolic genes, but also by the modulation of GA receptor *GID1* [21]. The upregulation of the GAI- and RGL2 DELLA-genes by GA observed in this study might also reflect feedback regulation. These results collectively suggest that GA can alter the expression of both metabolic genes and signaling components to achieve GA homeostasis in the apple shoot apex.

GA-responsive genes encoding transcription factors are potential candidates for mediating of the GA-mediated flowering pathway. Our finding that transcription factor-encoding, GA-responsive genes were overrepresented for functions in ‘cell differentiation’ and ‘response to GA’ is consistent with previous findings linking GAs with development, and further support that our data reflects a direct biological response to GA. Our finding that GA results in upregulation of two *KNAT1* homologs is particularly interesting. Class I KNOX transcription factors, including KNAT1 and STM (SHOOT MERISTEMLESS), may participate in GA signal transduction via regulation of a wide spectrum of downstream targets, including MADS-box genes, *GA20OX* and *GA2OX* [67]. More importantly, Arabidopsis KNAT1 mediates the balance of GA and CK in the shoot apical meristem via regulating their biosynthetic and catabolic genes [61, 68–70]. It was previously reported that a relatively low ratio of GA/CK in the apple shoot apex favors the formation of floral buds, whereas high GA/CK ratio favors vegetative buds [71], and it has been hypothesized that GA represses flowering in apple through antagonizing an inductive influence of CK [40]. Our observation that GA repressed expression of *AHP1*-like genes, which participate in transducing the CK signal, offers a potential mechanism for this.

We considered the possibility that, in apple, GA might directly repress expression of conserved floral promotive genes. In Arabidopsis, *SOC1* and *LFY* promote flowering as downstream targets of DELLA and GAMYB proteins, respectively [22–24]. We found no significant effects of GA on expression of homologous apple *SOC1* and *LFY* genes, consistent with previous findings of Fan et al. [31], although neither gene was strongly expressed in non-treated plants at this time in the season (**not shown**). Arabidopsis *FT* is expressed in the vasculature of leaves, and promotes flowering as protein trafficked to the shoot apex. In apple, *MdFT1* transcripts are expressed to appreciable levels directly in the apex, and we anticipated that GA might downregulate this expression. However, we found no significant effect of GA on *MdFT1* expression in the apex. Potentially, GA represses expression of *MdFT1* and/or *MdFT2* outside of the apex, with subsequent loss of *FT* trafficking.

A previous study reported that one of two apple *TFL1*-like genes, *MdTFL1–2*, was upregulated in the shoot apex of trees treated with GA_3_ [49], but this was apparent only well after GA treatment (30 DAT) and late in the season (64 DAFB) when flowers would be expected to be forming. During flowering in Arabidopsis, sustained expression of *TFL1* in the center of the inflorescence apex antagonizes expression of *LFY*, allowing for the generation of floral meristems in an indeterminate manner [52]. In apple, which produces a cyme-like, determinate inflorescence, production of the characteristic, terminal ‘King bloom’ may be associated with suppression of a *TFL1*-like activity in the inflorescence apex. If *MdTFL1–1* or *MdTFL1–2* have a role in meristem identity in apple similar to that of *TFL1* in Arabidopsis, then their relatively diminished expression in control, relative to GA-treated, plants late in the season might be an indirect consequence of flower formation. Nevertheless, the fact that transgenic suppression of *MdTFL1* in apple leads to early flowering strongly argues for an independent, direct role in flowering [51]. Possibly, the *MdTFL1* genes have both meristem identity and flowering functions. Our study revealed that both *MdTFL1–1 and MdTFL1–2* could be induced rapidly (within 1 DAT) by GA_4 + 7_, prior to flower formation, suggesting the regulation of *TFL1* genes by GA may be relatively direct. *TFL1* homologs have been well characterized in several perennial trees [72], but this is the first evidence that *TFL1* may modulate flowering as a downstream effector of GA.

## Conclusions

This transcriptional analysis of the response of the shoot apex to bioactive GA is a first step to identify molecular mechanisms directly connecting GA with flowering genes. Our study shows that the GA_4 + 7_ repressed flower formation without affecting shoot structure. Its application also induced the expression of a class of GA catabolic genes, *GA2ox*, via GA feedforward regulation, and repressed expression of GA receptors and GA transporters. Although previous experiments in other plants have shown similar regulation of GA signaling components by GA, this report reveals coordinated regulation of these multiple components. The identification of CK and other hormonal signaling components as GA responsive suggest that GA signaling interacts extensively with other hormonal signaling pathways. Our data supports several transcription factor candidates which may serve as regulators in GA-mediated repression of flowering in apple. Our study also strongly support the idea that GAs promote expression of repressive *TFL1*-like genes.

## Methods

### Plant material and growth conditions

Trees used in 2011 and 2018 experiments were located at the MSU Research Center in Clarksville, MI and were managed in accordance with standard commercial practices for disease, insect and weed control. The date of full bloom was defined as the date on which the maximum numbers of flowers were at anthesis.

Experiments using ‘Fulford Gala’ were carried out in 2011, and used trees established for 20 years as grafts onto ‘Mark’ rootstock. Six physiologically similar trees that showed 80–90% bloom density were chosen, and all flowers were removed by hand. For each tree, five branches, each between 4 cm and 6 cm in diameter at the base, were selected and randomly assigned for apex collection dates (four branches) or for observation of bloom density the following spring (one branch). Trees were randomly assigned as three replicate pairs, with each pair comprising one tree that was treated with GA approximately 35 days after full bloom (DAFB), and one plant that was treated on the same date with water containing surfactant only. GA was applied as a mixture of GA_4_ and GA_7_ (ProVide, Valent BioSciences) at 200 ppm (mg/l) active ingredient in aqueous solution containing 0.05% Tween-20 as surfactant. Replicate treatments were staggered by 1–2 d. Collections were made at 1, 2, 5, and 15 d after GA treatment (DAT), corresponding to 36, 37, 40, 50 DAFB. On each collection date, one or two dominant buds immediately subtending the position of the inflorescence, or the apex of actively growing shoots originating from this position, were dissected using a razor blade, immediately frozen in liquid nitrogen, and stored at − 80 °C. Buds and apices were less than 4 mm in diameter and included developing leaves less than 3 mm in length. Flower formation was evaluated at full bloom in the following year.

Experiments using ‘Brookfield Gala’ were carried out in 2018 and used 36 six-year-old trees as grafts onto ‘M9-Pajam 2’ rootstocks. Uniform trees were selected and divided into three groups for treatment with GA_3_ or GA_4 + 7_, or to serve as a control. Each group comprised three biological replicates with each replicate including four Gala trees. Shortly after full bloom, physiologically similar spurs were identified and tagged for study. For each replicate, 15 spurs were designated for sampling for each of the four time points, that is 5, 10, 28, and 50 DAT, corresponding to 35, 40, 58 and 80 DAFB. Gibberellins were applied using a pressure sprayer at 30 days after full bloom (June 13 2018) until leaves were drenched. GA_4 + 7_ was applied as a mixture of GA_4_ and GA_7_ at a concentration of 200 ppm; GA_3_ was applied at a concentration of 300 ppm; water was applied in the control. Around 0.1% Regulaid was used as surfactant for each spray. Tagged spurs were collected in the field and brought back the lab in a hydrated cooler maintained at ~15C. The first fully expanded bourse shoot leaves (around 2 mm at the one third base of leaves) and the adjacent bourse shoot apices were dissected from the spurs, frozen immediately in liquid nitrogen, and subsequently stored at − 80 °C until use.

### Nucleic acid preparation, sequencing, and data analyses

RNA was isolated from frozen apex samples using the method of Gasic et al. [73], with the exception that spermine was substituted for spermidine in the extraction buffer, followed by a final ‘clean-up’ step using a commercial kit (RNeasy Mini; Qiagen). Library preparation and sequencing used the Illumina platform and TruSeq 101-b, paired-end protocols, starting with 1 μg of total RNA from each sample. The raw sequence files were filtered with fastq-mcf [74], using the parameters -t 0.10 -p 15 -l 20 -q 25, to remove adapter sequences, reads less than 20 bases long, and terminal bases with a Phred score below 25. A census of filtered sequences is shown in Additional file 1.

Analyses of RNA-seq data were conducted through a pipeline comprising several off-the-shelf programs, with modifications as follows. Sequences were mapped to the GDDH13 genome sequence [56] using HISAT 2.1 [75] and assembled into transcriptional models using StringTie (version 1.3.3) [76] with default parameters, including the -G option for use of a reference annotation as outlined in [77]. A merged transcriptome representing all libraries was generated with the “merge” argument in StringTie. Sequences of all transcript models are shown in Additional file 2, and genomic coordinates are shown in Additional file 6. Transcripts that showed positional overlap with genes and/or exons previously annotated in the reference genome were identified using the Gffcompare program included in the Cufflinks suite [78]. For these, the reference gene ID was used. Transcripts showing no overlap with annotated exons were defined as novel; these included those that were intergenic, fully contained within a reference intron, or that contained a reference gene within their intron(s). Novel transcripts were analyzed for homology with known sequences using the Blastn module of the Basic Local Alignment Search Tool Suite (Version 2.4.0+) and the NCBI (National Center for Biotechnology Information) non-redundant nucleotide (nt) database (updated on June 8, 2018) [79] with an Expect (E) value cutoff of 1e-12.

Loci expression levels and differences in expression were estimated using programs within the Cufflinks suite [78] as follows. Transcript models were analyzed for read representation using Cuffquant, and FPKM values were normalized for library size using Cuffnorm. Significant differences in representation among samples were determined using Cuffdiff. Differentially expressed genes (DEGs) were designated as significant using an adjusted *P*-value (Benjamini–Hochberg adjustment) and a false discovery rate of 0.05. All transcript models were analyzed for homology with Arabidopsis protein models using the Basic Local Alignment Search Tool (BLAST) [80], blastx (BLAST 2.7.1+), and The Arabidopsis Information Resource (TAIR) 10 genome open reading frame translations and annotations (TAIR10_pep_20101214_updated), with an E-value cutoff of 1e-12. For each apple locus showing a match, a single representative transcript model was reported in Additional file 9 based on the highest bitscore. An apple counterpart of a given Arabidopsis protein was defined as the transcript(s) that showed highest homology to that protein, respective to all other proteins in the TAIR dataset. GO bioprocess and KEGG pathway enrichment analysis used online DAVID tools [81] and were based on the corresponding TAIR annotation. TAIR IDs for Arabidopsis homologs that encode transcription factors were obtained from Plant TFDB [82].

### *Cis*-element enrichment analysis

The 500-bp region upstream of the translation start site was analyzed using tools within the MEME suite [83]. To cover both short and long sequence patterns, DREME (command-line version) [84] was conducted in the discriminative mode to identify relatively short (6–8 bp) motifs, while MEME (command-line version) [85] was used to discover relatively long (up to 20 bp) sequence motifs. Promoters of all genes expressed (FPKM > 0) at the same time point were used as control sequences (*n* > 40,000). Identified enriched motifs were compared with known motifs cataloged in two Arabidopsis motif databases (DNA affinity purification sequencing [62]; protein-binding microarrays [63]) using TOMTOM [86].

### TaqMan® primer and probe design

TaqMan® gene expression assays were conducted to confirm the expression of the most significant genes of interest, including *GA2OX*, *NPF3.1, GID1*, *TFL1*, *FT1* and *FT2*. To design the primers and probes specific for the target sequences, the target sequences were used as query to blast against apple nucleotide collection database (taxid: 3750) using Blastn [80]. They were then aligned with their homologs using SnapGene 2.3.2. The primers and probes (Additional file 16) were designed based such that at least one sequence of each primer-probe set was stretched over the exon junction and was specific to their target rather than any other sequences. Adenine (A) at 3′ end of the primers was avoided. Each primer was 20–30 bp long and the GC content varied from 30 to 60% and the optimal melting temperatures from 55 °C to 62 °C. TaqMan® MGB (Minor Groove Binder) probes for Custom TaqMan® Gene Expression Assay were designed 1–2 bp away from the 3′-terminal regions of the forward primers (the upstream primer) with A, T (Thymine) or C (Cytosine) as the first nucleotide, a high C/G (Guanine) ratio and a length of 15–18 bp. The length of amplicons for Custom TaqMan® Gene Expression Assay was less than 150 bp. The specificity of the primers was confirmed by Primer-BLAST [87] using the template sequences and our own primers as query. The self-complementarity and 3′ self-complementarity of these primers varied from 0 to 6. The absolute ΔG(kcal.mol^− 1^) value for hairpin was limited under 2. The absolute ΔG value for self-dimer and hetero-dimer was less than 9. Primers and probe for the apple internal control gene *ACTIN* were designed following similar rules, except for that *ACTIN* probe was labeled with VIC dye. *ACTIN* primers and probe were added into every reaction system with those of genes of interest to ensure accuracy.

### Taqman qRT-PCR

Two-step Quantitative Real-Time Polymerase Chain Reaction (qRT-PCR) was conducted to confirm the expression of interesting genes using and qPCR machine (Agilent Technologies Stratagene Mx3005P). Specifically, TaqMan™ Gene Expression Master Mix (10 μl), 5x diluted cDNA template (2 μl), *ACTIN* forward (1 μl) and reverse (1 μl) primers, *ACTIN* probe (1 μl), primer-probe assay of genes of interest (1 μl), and ddH2O (4 μl) were added into microtubes and mixed well gently. The thermal profile used for Taqman qPCR reactions was set according to the guide (Introduction to Quantitative PCR) provided by Agilent Technologies: product was melt at 95 °C for 15 s, followed by 40 cycles of primer annealing and Taq DNA polymerase extension at 60 °C for 60 s. ROX was set as the passive reference dye.

## Supplementary information

**Additional file 1: Table S1.** Quantity and mapping rates for RNA-seq data generated for each sample.

**Additional file 2.** Sequence of apple shoot apex transcript models, based on alignment of sequence reads to the GDDH13 reference genome and assembly of aligned reads.

**Additional file 3.** Estimated expression of individual transcript models identified in this study in the GDDH13 reference genome. Expression is presented as FPKM (Fragments Per Kilobase of transcript per Million Mapped reads).

**Additional file 4.** Estimated expression of loci identified in this study.

**Additional file 5: Table S2.** Classification of transcript models using GffCompare. Class codes were assigned to each transcript based on the relationship between this transcript and its closest reference transcript. **Table S3.** Best hits for unannotated transcripts (class codes: i, y, p, u) from BlastN and the NCBI non-redundant nucleotide collection database (nt). **Table S4.** Sequence length and expression of unannotated transcripts that have no hit from the nt database.

**Additional file 6.** A revised feature coordinate map (GFF3) including previously annotated transcripts and transcripts identified in this study.

**Additional file 7: Table S5.** Differentially expressed genes in the apple bourse shoot apices in response to GA. This file contains the differentially expressed genes (DEGs) in response to GA, their coordinates, Arabidopsis homologs, gene description, expression (FPKM), Log2(Fold Change), and significance (*p* value, and q value, which is adjusted *p* value).

**Additional file 8. **Enriched GO bioprocess terms (**Table S6**) and KEGG pathway terms (**Table S7**) for GA-responsive genes. Gene Ontology bioprocess and KEGG pathway enrichment analysis were carried out using annotated terms for the most homologous Arabidopsis protein. All Arabidopsis homologs of apple genes supporting the enrichment terms are listed. GA-related terms are highlighted in red. The *P* values for enrichment, as well as the Bonferroni and Benjamini corrected *p* values are shown.

**Additional file 9: Table S8.** Results of Blastx using transcript models and the Arabidopsis TAIR10 non-redundant protein database.

**Additional file 10: Table S9.** DEGs in the enriched plant hormone pathways based on KEGG pathway analysis. Color scales indicate upregulation (red) or downregulation (blue) of DEGs by GA.

**Additional file 11: Figure S1.** *MdFT1* is preferably expressed in the bourse shoot apex, compared with *MdFT2*. A: Read counts for *MdFT1* and *MdFT2*. Read counts were obtained from the library C5, which is RNA-seq data of CON shoot apex samples collected at 36 DAFB in 2011. B: Expression of *MdFT1* and *MdFT2* in shoot apex and bourse shoot leaf. Blue columns represent expression of *MdFT1*; pink columns represent expression of *MdFT2*. RT-PCR data was relative expression obtained from shoot apex on fruiting spurs at 33 DAFB in 2017.

**Additional file 12: Figure S2.** GA treatment does not affect the expression of *MdFT1* in the bourse shoot apex. Data was obtained from the 2018 experiments. Green line represents the expression in GA_4 + 7_-treated samples, while black dash line represents CON. Double time scales are given on Y axis: DAFB, days after full bloom; DAT, days after treatment.

**Additional file 13: Table S10.** DEGs that potentially encode transcription factors.

**Additional file 14: Figure S3.** GO bioprocess enrichment analysis for DEGs that potentially encode transcription factors. GO bioprocess terms listed are all significant enriched (Benjamini-adjusted p value < 0.05). Fold enrichment is the enrichment magnitude of test set compared with reference.

**Additional file 15: Figure S4.** Enriched motifs or potential binding sites for transcription factors. Motifs with a p value less than 0.05 were considered to be significantly enriched compared to the control sequences. Listed motifs were enriched at 2 DAT, except for motifs for LMI1 (5 DAT) and RVE1 (15 DAT). Short motifs (less than 8 bp) that were discovered by DREME and long motifs (15–20 bp) by MEME.

**Additional file 16: Table S11.** Taqman primers and probes for genes of interest and internal control gene (*ACTIN*).

## Data Availability

All data generated or analyzed during this study are included in this published article and its supplementary information files. The datasets generated and/or analysed during the current study are available in the NCBI Sequence Read Archive (SRA) (accession number: PRJNA299491), https://www.ncbi.nlm.nih.gov/bioproject?LinkName=sra_bioproject&from_uid=2005070.

## Abbreviations

ABA: Abscisic Acid
AP1: APETALA1
bZIP: BASIC LEUCINE ZIPPER transcription factor(s)
CDF: CYCLING DOF FACTOR
CK: cytokinin
CPS: ENT-COPALYL DIPHOSPHATE SYNTHASE
DAT: Days after Treatment
EUI: ELONGATED UPPERMOST INTERNODE
FPKM: Fragments Per Kilobase of transcript per Million mapped reads
FT: FLOWERING LOCUS T
GA: Gibberellin
GA20OX: GA20 OXIDASE
GA2OX: GA2 OXIDASE
GA3OX: GA3 OXIDASE
GAI: GIBBERELLIC ACID INSENSITIVE
GID1: GIBBERELLIN-INSENSITIVE DWARF 1
GO: Gene Ontology
KNAT1: KNOTTED-LIKE FROM *ARABIDOPSIS THALIANA* 1
KO: ENT-KAURENE OXIDASE
KS: ENT-KAURENE SYNTHASE
LFY: LEAFY
LMI1: LATE MERISTEM IDENTITY 1
MYB: MYELOBLASTOSIS family of transcription factors
NPF: NITRATE TRANSPORT 1/PEPTIDE TRANSPORTER FAMILY
RGL2: REPRESSOR OF GA-LIKE 2
RVE: REVEILLE
SOC1: SUPPRESSOR OF OVEREXPRESSION OF CO 1
STM: SHOOT MERISTEMLESS
TCP14: TEOSINTE BRANCHED1/ CYCLOIDEA/PROLIFERATING CELL FACTOR 14
TFL1: TERMINAL FLOWER1
TOE1: TARGET OF EARLY ACTIVATION TAGGED 1
