# Supplementary figures and images for: Genetic mechanisms in the repression of flowering by gibberellins in apple (Malus x domestica Borkh.)
Source: BMC Genomics. 2019 Oct 16;20:747. doi: 10.1186/s12864-019-6090-6 (PMC6796362; doi:10.1186/s12864-019-6090-6)

A

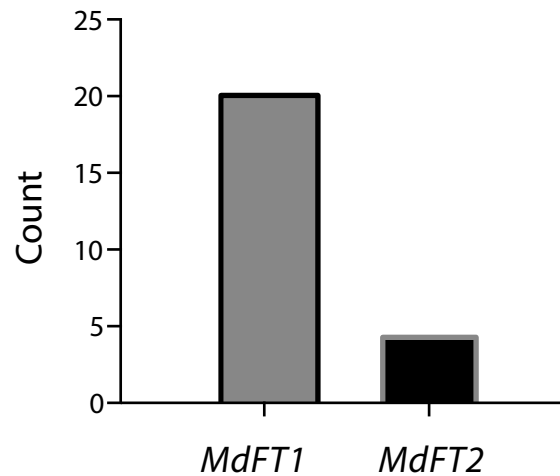

B

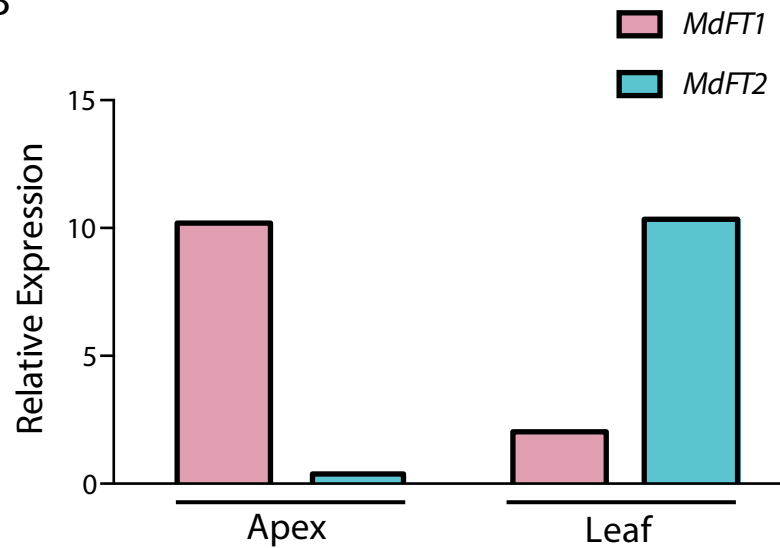

Supplement: Supplementary file 11 — Additional file 11: Figure S1. MdFT1 is preferably expressed in the bourse shoot apex, compared with MdFT2. A: Read counts for MdFT1 and MdFT2. Read counts were obtained from the library C5, which is RNA-seq data of CON shoot apex samples collected at 36 DAFB in 2011. B: Expression of MdFT1 and MdFT2 in shoot apex and bourse shoot leaf. Blue columns represent expression of MdFT1; pink columns represent expression of MdFT2. RT-PCR data was relative expression obtained from shoot apex on fruiting spurs at 33 DAFB in 2017. [file 12864_2019_6090_MOESM11_ESM.pdf]

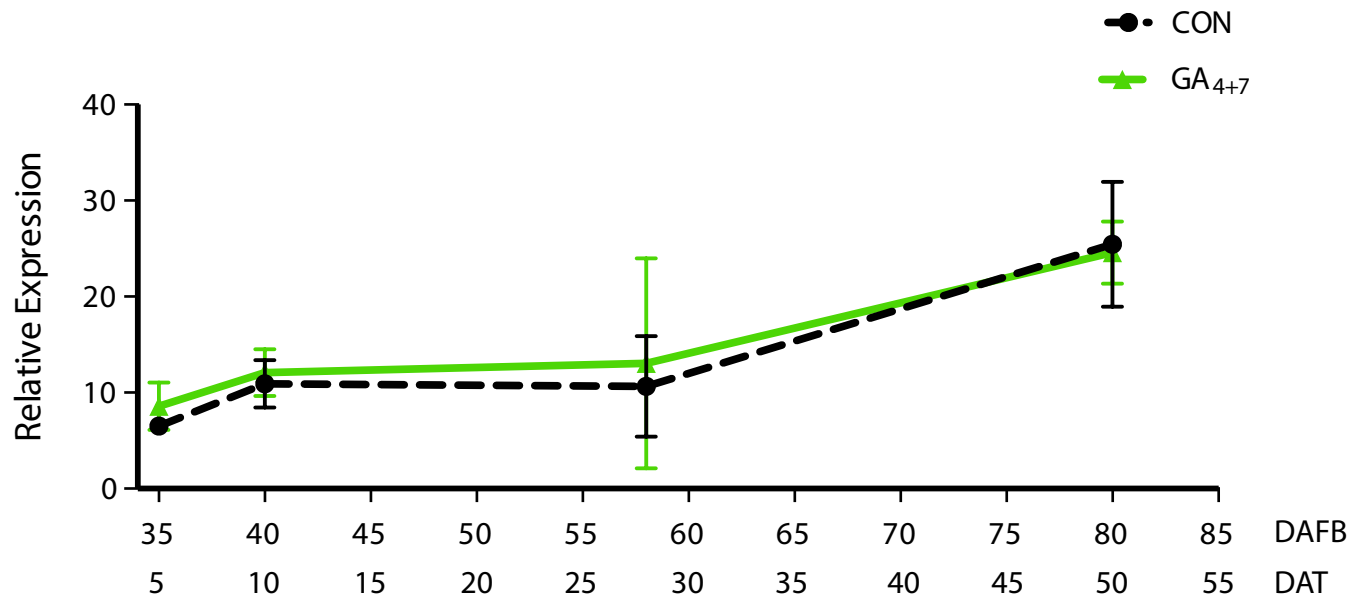

Supplement: Supplementary file 12 — Additional file 12: Figure S2. GA treatment does not affect the expression of MdFT1 in the bourse shoot apex. Data was obtained from the 2018 experiments. Green line represents the expression in GA4 + 7-treated samples, while black dash line represents CON. Double time scales are given on Y axis: DAFB, days after full bloom; DAT, days after treatment. [file 12864_2019_6090_MOESM12_ESM.pdf]

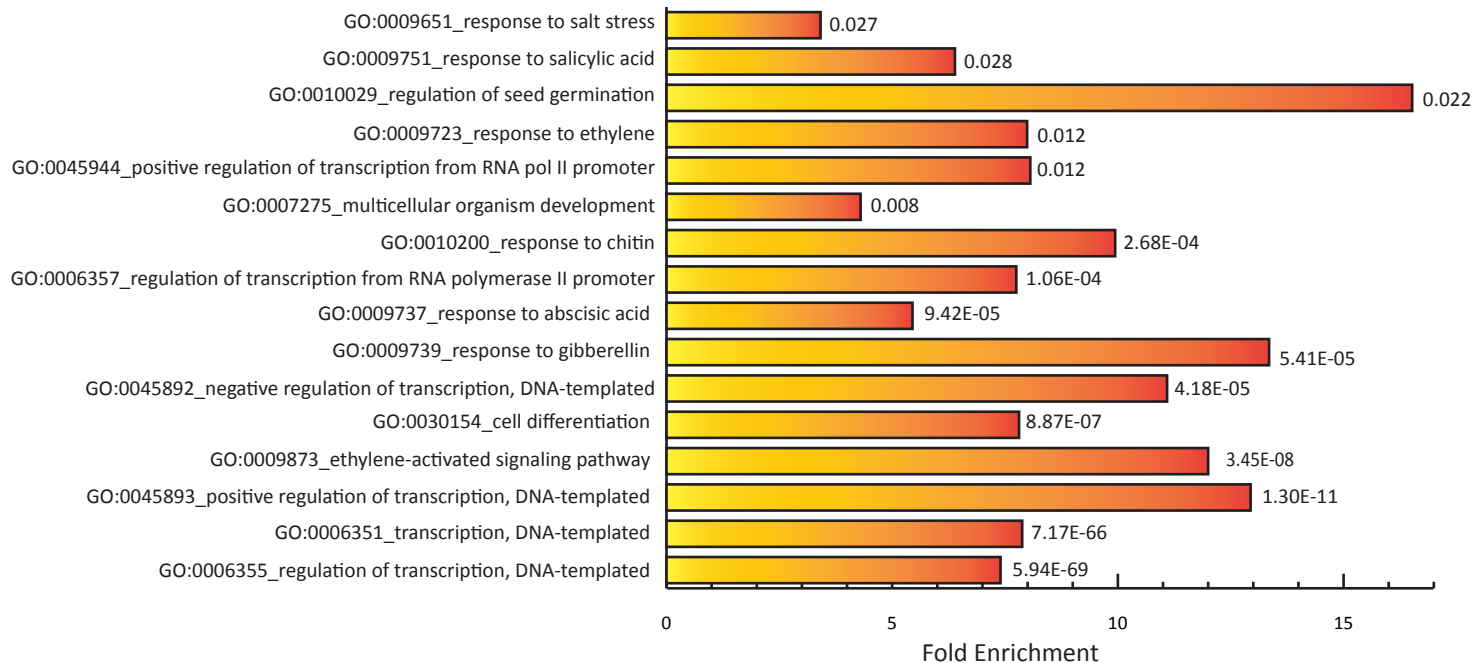

Supplement: Supplementary file 14 — Additional file 14: Figure S3. GO bioprocess enrichment analysis for DEGs that potentially encode transcription factors. GO bioprocess terms listed are all significant enriched (Benjamini-adjusted p value < 0.05). Fold enrichment is the enrichment magnitude of test set compared with reference. [file 12864_2019_6090_MOESM14_ESM.pdf]

# Transcription Factor

# Enriched motif

# Binding site

DEL1/2

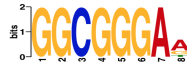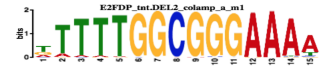

EIN3

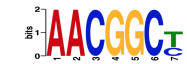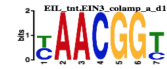

FUS3

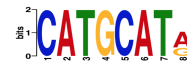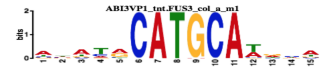

bZIP42/44

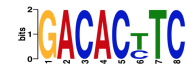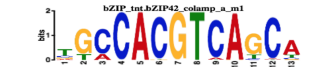

MYB1

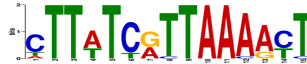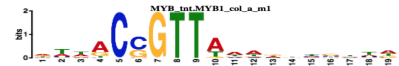

WRKY14

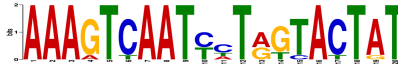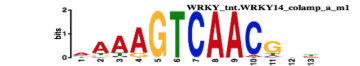

CDF3/5

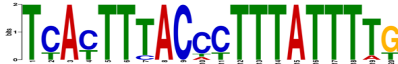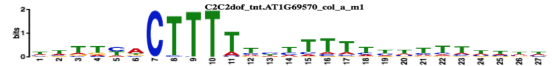

bZIP44/48

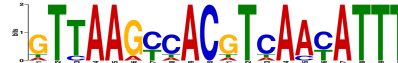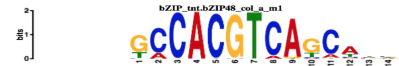

REM1

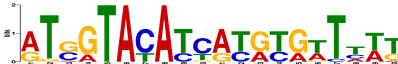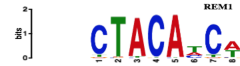

LIM1

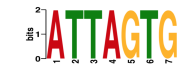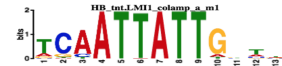

RVE

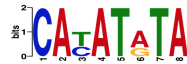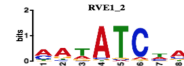

Supplement: Supplementary file 15 — Additional file 15: Figure S4. Enriched motifs or potential binding sites for transcription factors. Motifs with a p value less than 0.05 were considered to be significantly enriched compared to the control sequences. Listed motifs were enriched at 2 DAT, except for motifs for LMI1 (5 DAT) and RVE1 (15 DAT). Short motifs (less than 8 bp) that were discovered by DREME and long motifs (15–20 bp) by MEME. [file 12864_2019_6090_MOESM15_ESM.pdf]
